# LncRNA FBXL19-AS1 promotes proliferation and metastasis of cervical cancer through upregulating COL1A1 as a sponge of miR-193a-5p

**DOI:** 10.1186/s40709-021-00151-8

**Published:** 2021-08-16

**Authors:** Xiaoyong Huang, Haiyan Shi, Xinghai Shi, Xuemei Jiang

**Affiliations:** 1 Department of Medical Laboratory, Medical College of Yan’an University, Yan’an, 716000 Shaanxi China; 2 Department of Laboratory, The First People’s Hospital of Urumqi, Ürümqi, 830000 Xinjiang China; 3 Department of Laboratory, Xinjiang Uygur Autonomous Region Maternal and Child Health Hospital, No. 1 Renmin Road, Ürümqi, 830000 Xinjiang People’s Republic of China

**Keywords:** Cervical cancer, Proliferation, Metastasis, FBXL19-AS1, miR-193a-5p, COL1A1

## Abstract

**Background:**

Cervical cancer (CC) is one of the most common and malignant tumors in women. In this study, we aim to explore the role and mechanism of F-box and leucine rich repeat protein 19 antisense RNA 1 (FBXL19-AS1), a novel long-chain non coding RNA (lncRNA) with marked roles in a variety of tumors, in regulating the proliferation and metastasis of CC.

**Methods:**

The expression of FBXL19-AS1, miR-193a-5p and COL1A1 were detected by RT-PCR and western blot. Gain- and loss-of functional assays of FBXL19-AS1 and miR-193a-5p were performed in CC cell lines in vitro or in vivo. The proliferation, migration, invasion, apoptosis and epithelial-mesenchymal transition (EMT) of CC cells were determined.

**Results:**

FBXL19-AS1 and COL1A1 were significantly up-regulated in CC tissues, while miR-193a-5p was significantly down-regulated. Overexpression of FBXL19-AS1 significantly promoted the proliferation, migration, invasion, EMT and growth of CC cells and inhibited apoptosis, while knockdown of FBXL19-AS1 had the opposite effects. On the other hand, miR-193a-5p inhibited the proliferation and metastasis of CC cells. Mechanistically, FBXL19-AS1 functioned as a competitive endogenous RNA (ceRNA) and inhibited the expression of miR-193a-5p, which targeted at the 3’-UTR site of COL1A1 and negatively regulated COL1A1 expression.

**Conclusions:**

FBXL19-AS1 promotes the proliferation and metastasis of CC cells by sponging miR-193a-5p and up-regulating COL1A1.

## Background

There are many types of cancers in the female genital system; cervical cancer (CC) is one of the most prevailing malignancies in the female reproductive tract, with high morbidity and mortality in female tumors [1]. At present, the main clinical treatments for CC include surgery, radiotherapy and chemotherapy. However, there are no specific clinical symptoms in the early stage of CC, and many patients are in an advanced stage at the time of consultation. Additionally, the indications for surgical treatment are limited by tumor staging, and the severe side effects of radiotherapy and chemotherapy have harmful impacts on womens’ psychophysiology [2]. Therefore, it is urgent to establish a more effective strategy for early diagnosis and targeted therapy.

Long non-coding RNAs (lncRNAs) are non-coding RNAs with more than 200 nt in length and function as gene expression regulators that play a vital role in regulating cell metabolism and growth [3]. In addition, lncRNAs affects the apoptosis, invasion and metastasis of tumor cells and have a significant influence on tumor development [4]. The altered lncRNAs in tumors are expected to be used as diagnostic markers in multiple tumors, such as lncRNA HOTAIR (in breast cancer) [5] and lncRNA MALAT1 in liver cancer, breast cancer, non-lung small cell carcinoma, colon cancer and prostate cancer [6]. LncRNA F-box and leucine rich repeat protein 19 antisense RNA 1 (FBXL19-AS1), with a length of 3951 bp, is located in human chromosome 16. Moreover, FBXL19-AS1 plays a role in tumor progression. For example, FBXL19-AS1 expression is remarkably increased in breast cancer and promotes its development via promoting proliferation and reducing cell apoptosis [7]. Some researchers also stated that FBXL19-AS1 promotes the growth, metastasis and invasion of osteosarcoma by targeting miR-346 [8]. However, the functions and mechanisms of lncRNA FBXL19-AS1 in cervical cancer are still elusive.

MicroRNAs (miRNAs), as a non-coding RNA with 20 ~ 22 nt in length, can implement in the degradation of the targeted mRNA, thus regulating gene expression in a post-transcriptional level and exerting a regulatory effect in the biological behaviors of tumor cells [9]. MiR-193a-5p has been proven its participation in the biological processes of various tumors. For example, miR-193a-5p is highly expressed in hepatocellular carcinoma [10], lung cancer [11] and prostate cancer [12], and plays a carcinogenic role. However, it has anti-tumor effects on gastric cancer [13], osteosarcoma [14] and breast cancer [15] with lower expression in those tumors. Unfortunately, there are few reports about miR-193a-5p in cervical cancer.

Collagen type I alpha 1 chain (COL1A1) is a gene located at 17q21.33 and encodes the pro-alpha1 chains of type I collagen whose triple helix comprises two alpha1 chains and one alpha2 chain [16]. COL1A1 has been also reportedly involved in the development of many human diseases such as sports-related tendon and ligament injuries [17] and osteogenesis imperfecta [18]. Interestingly, COL1A1 is proved to be candidate prognostic factor in several cancers such as gastric cancer [19]. Others demonstrate that COL1A1 is increased in CC tissues and COL1A1 activation could inhibit the apoptosis of CC cells [20]. It can be deduced that COL1A1 gene is related to the biological characteristics of CC while the upstream mechanism of COL1A1 in CC remains to be explored.

Notably, the “competing endogenous RNA (ceRNA)” model suggests that lncRNAs can act as ceRNAs containing miRNA-binding sites, sponge their targeted miRNAs. Therefore, lncRNAs affect miRNA-mediated post-transcriptional gene regulation [21, 22]. Interestingly, this ceRNA mechanism has also been discovered in tumors [23]. Here, FBXL19-AS1 expression was found to be increased in CC tissues and associated with poorer overall survival of CC patients. Further experiments confirmed that FBXL19-AS1 promotes the proliferation, metastasis and invasion of CC cells. Additionally, FBXL19-AS1, as the ceRNA on miR-193a-5p, was found to upregulate COL1A1 expression. Therefore, we suggest that FBXL19-AS1 can modulate the progression of CC dependently through the miR-193a-5p/COL1A1 axis.

## Methods

### Tissue specimens

Forty-six cervical cancer patients who underwent surgical treatment in Xinjiang Uygur Autonomous region Maternal and Child Health Hospital from October 2014 to October 2015 were selected. No radiotherapy or chemotherapy or other anti-tumor treatment was given to the patients before surgical treatment. The patients had an average age of 52 years, with the youngest aged 32 and the oldest 69 years. All subjects in this study have signed informed consent, and the research ethics committee of Xinjiang Uygur Autonomous region Maternal and Child Health Hospital approved the study.

### Cell culture

Human healthy cervical cells (HUCEC) and human cervical cancer cells (HeLa, Caski, C-33 A, AV3) were purchased from the American Type Culture Collection (Rockville, MD, USA). The cells were cultured in an RPMI1640 (Thermo Fisher Scientific, MA, USA) medium containing 10% fetal bovine serum (FBS) (Thermo Fisher Scientific, MA, USA) and 1% penicillin/streptomycin (Invitrogen, CA, USA) at 37 ℃ with 5% CO_2_ volume fraction. During the logarithmic growth phase, 0.25% trypsin (Thermo Fisher HyClone, Utah, USA) was used for cell trypsinization. We selected C-33 A and Hela cells as auxiliary research objects since their large expression differences.

### Cell transfection

C-33 A and HeLa cells (at the logarithmic growth stage) were trypsinized by 0.25% trypsin and transited into 6-well plates (5 × 10^6^ per well). After cell growth was stable, they were transfected with the over-expressed FBXL19-AS1 plasmids, small inference RNA targeting FBXL19-AS1 (si-FBXL19-AS1) or COL1A1 (si-COL1A1), miR-193a-5p mimics, miR-193a-5p inhibitor [anti-miR193a-5p and their negative controls according to the instructions of FuGENE® HD Transfection Reagent (Roche, Shanghai, China)]. At 37 ℃ with 5% CO_2_, the cells were cultured in an incubator. After 24 h of transfection, the medium was changed with fresh compete medium and the cells were kept incubated for another 48 h. The total RNA of the cells was extracted for real-time fluorescent quantitative PCR (RT-PCR) to detect the transfection efficiency. After successful transfection, they were used in subsequent experiments.

### RT-PCR

TRIzol method was performed to extract total RNA from CC tissues and cells routinely, and the extracted total RNA was used to remove genomic DNA by deoxyribonuclease I (Sigma-Aldrich, St. Louis, Missouri, USA). The reverse transcription reaction (for FBXL19-AS1 and COL1A1) was carried out according to the operation procedure of the reverse transcription kit (Thermo, Shanghai, China), and the reaction conditions were: 70 ℃, 10 min; 5 min on ice; 42 ℃, 60 min; 95 ℃, 5 min; 0 ℃, 5 min. For the cDNA synthesis of miR-193a-5p, a commercially purchased HyperScript III miRNA 1st Strand cDNA Synthesis Kit (by stem-loop) (Cat.No.R601, NovaBio, Shanghai, China) was used, and all procedures were performed according to the instructions of the manufacturer. The fluorescence quantitative PCR reaction system was 25 µL, containing 500 ng cDNA template, 250 nmol L^−1^ reverse and forward primers, and 12.5 µL of 2×SYBR Green PCR Master mixture (Solarbio, Beijing, China). β-actin and U6 were used as the endogenous control. Primers for FBXL19-AS1: forward 5′-CCCATTGTCCCCATTTTGCA-3′, reverse 5′-AGGCAGCAGGAATCAGTCTT-3′; miR-193a-5p primers: forward 5′-CAGTGCAGGGTCCGAGGT-3′, reverse 5′-AACAATTGGGTCTTTGCGGGC-3′; primers for COL1A1: forward 5′-CCTGGATGCCATCAAAGTCT-3′, reverse 5′- AATCCATCGGTCATGCTCTC-3′; primers for β-actin: forward 5′-CTCCATCCTGGCCTCGCTGT-3′, reverse 5′-CCAAGGAGTAAGACCCCTGG-3′; primers for U6, forward 5′-TCTTCGTCATCACATATACTAAAAT-3′, reverse 5′-CTCTTCACGAATTTTCGTGTCAT-3′. The reaction tube was placed into the MX3000P Real-time PCR reaction instrument (Agilent, California, USA), and the reaction conditions were: 94 °C, 55 °C, 72 °C, 45 cycles, with fluorescence signal monitoring. The 2^(−ΔΔCt)^ value indicates the relative expression of genes, and Ct value represents the number of amplification cycles passed when the fluorescence signal of the amplified product reached the set threshold during the PCR amplification process. Δ Δ process sample under test (Ct target gene − Ct β-actin)-in the control group (Ct target gene − Ct β-actin).

### Cell counting kit-8 (CCK-8) method

C-33 A and HeLa CC cells in the exponential growth phase were taken and made into a single-cell suspension. After being counted, cell density was adjusted as 1000 cells per well. We inoculated them in 96-well plates (6 replicates in each group, 6 well plates in total) and cultured for 12 h, 24 h, 48 h, 72 and 96 h. After adherent culture, with the addition of 90 µL medium and 10 µL CCK-8 solution (Beyotime, Shanghai, China) into the samples, blank control wells containing only CCK-8 solution and medium were set. After a 2-h incubation, the absorbance (A) value of each well was evaluated and recorded with Multiskan FC Microplate Reader (Thermo Fisher, MA, USA) at the wavelength of 450 nm.

### Western blot

We applied the Total Protein Extraction [Cat. no. C006225-0050, Sangon Biotech (Shanghai) Co., Ltd.] to extract the total proteins of different cell groups. BCA method (Beyotime, Shanghai, China) was applied for protein concentration SDS-PAGE electrophoresis was taken to isolate the total protein with 50 µg total proteins per pore. After 2-h electrophoresis, the proteins were wet transferred to PVDF membranes, sealed with 5% skim milk powder (1 h) and incubated with primary antibodies of Anti-Bax antibody (ab32503, 1:1000, Abcam, USA), Anti-bcl2 antibody (ab59348, 1:1000, Abcam, USA), Anti-Cleaved Caspase3 antibody (ab2302, 1:1000, Abcam, USA), Anti-E-Cadherin antibody (ab40772, 1:1000, Abcam, USA), Anti-Vimentin antibody (ab92547, 1:1000, Abcam, USA), Anti-SNAIL antibody (ab53519, 1:1000, Abcam, USA), Anti-COL1A1 antibody (39,952, Cell Signaling Technology, USA), Anti-beta Actin antibody (ab8227, 1:1000, Abcam, USA) overnight at 4 ℃. The next morning, the membranes were rinsed with TBST and incubated at 37 ℃ with the addition of horseradish peroxidase-labeled Goat Anti-rabbit (ab6721, 1:300, Abcam, USA) (1 h). The protein bands were exposed by Chemistar™ High-sig ECL Western Blotting Substrate (TANON SCIENCE & TECHONLOGY CO., Shanghai, China). The experiment was repeated three times.

### Transwell assay

The C-33 A and HeLa CC cells were seeded into the Transwell chambers (2.5 × 10^4^ cells per well) 24 h after transfection. The cells in the upper chambers were resuspended in a serum-free medium and the lower chambers were added with medium containing 20% serum. For invasion experiments, a layer of Matrigel (Sigma-Aldrich, St. Louis, Missouri, USA) was laid inside the chamber to simulate extracellular matrix (final concentration 2 mg mL^−1^, diluted with serum-free medium, 40 µL each chamber, 37 ℃, 30 min to 1 h until gelation), while for migration experiments, no Matrigel was added to the chamber. After 24-h cell migration and invasion, the cells were removed, secured with a mixture of formaldehyde and acetic acid for 15 min, rinsed with PBS, stained with crystal violet, and washed with 1 × PBS after staining. Finally, the cells in the chamber were wiped off with cotton swabs, and the quantity of those cells invaded and migrated were counted under the microscope.

### Dual luciferase activity experiment

All luciferase reporter vectors (FBXL19-AS1-MT, FBXL19-AS1-WT, COL1A1-MT, COL1A1-WT) were constructed by Promega (Madison, WI, USA). C-33 A cells (4.5 × 10^4^) were inoculated in a 48-well plate and cultured till 70% confluence. Then, we used lipofectamine 2000 to co-transfect those luciferase reporter vectors with miR-193a-5p mimics or negative control in C-33 A cells. Forty-eight hours after transfection, the luciferase activity was detected following manufacturer’s instructions of the dual luciferase reporter assay system (Promega, Wisconsin, USA). All experiments were made in triplicate and repeated three times.

### Animal experiments

For evaluating the role of FBXL19-AS1 on the growth of CC cells, 5 × 10^6^ C-33 A cells transfected FBXL19-AS1 overexpression plasmids or the negative control (NC) were subcutaneously injected into the right side of Balb/c nude mice (6-week-old, 22–25 g). During the next four days of incubation, the tumor volume was measured every week and calculated (the volume calculating formula: 0.5 × length × width^2^). At the 28th day, the mice got anaesthesia (Phenobarbital sodium, 50 mg kg^−1^) and sacrificed. Then the formed tumors were weighted and subjected to immunohistochemistry for detecting the expression of E-cadherin, Vimentin and Snail referred to [24]. All nude mice were fed in a specific pathogen free (SPF) environment free for food and water (12-dark and 12-night in a cycle, 25 ℃, 50% humidity). All animal experiments were conducted following national and international guidelines and approved by the Animal Ethic Committee of Xinjiang Uygur Autonomous region Maternal and Child Health Hospital.

### Data analysis

We applied SPSS17.0 statistical software (SPSS Inc., Chicago, IL, USA) to analyze the results. Measurement data were presented as mean ± standard deviation (x ± sd). We employed the t-test for the mean of two sample groups and the one-way analysis of variance (ANOVA) followed by Tukey post hoc test for the analysis of multiple samples. Pearson’s Correlation Analysis analyzed the correlation between the FBXL19-AS1 and miR-193a-5p level in CC tissues. Chi square test was used for analyzing the relationship of FBXL19-AS1 with the clinicopathological features of CC patients. *p* < 0.05 was considered to be statistically valuable.

## Results

### FBXL19-AS1 was upregulated in CC tissues and modulated CC cell proliferation and apoptosis

**Fig. 1:**
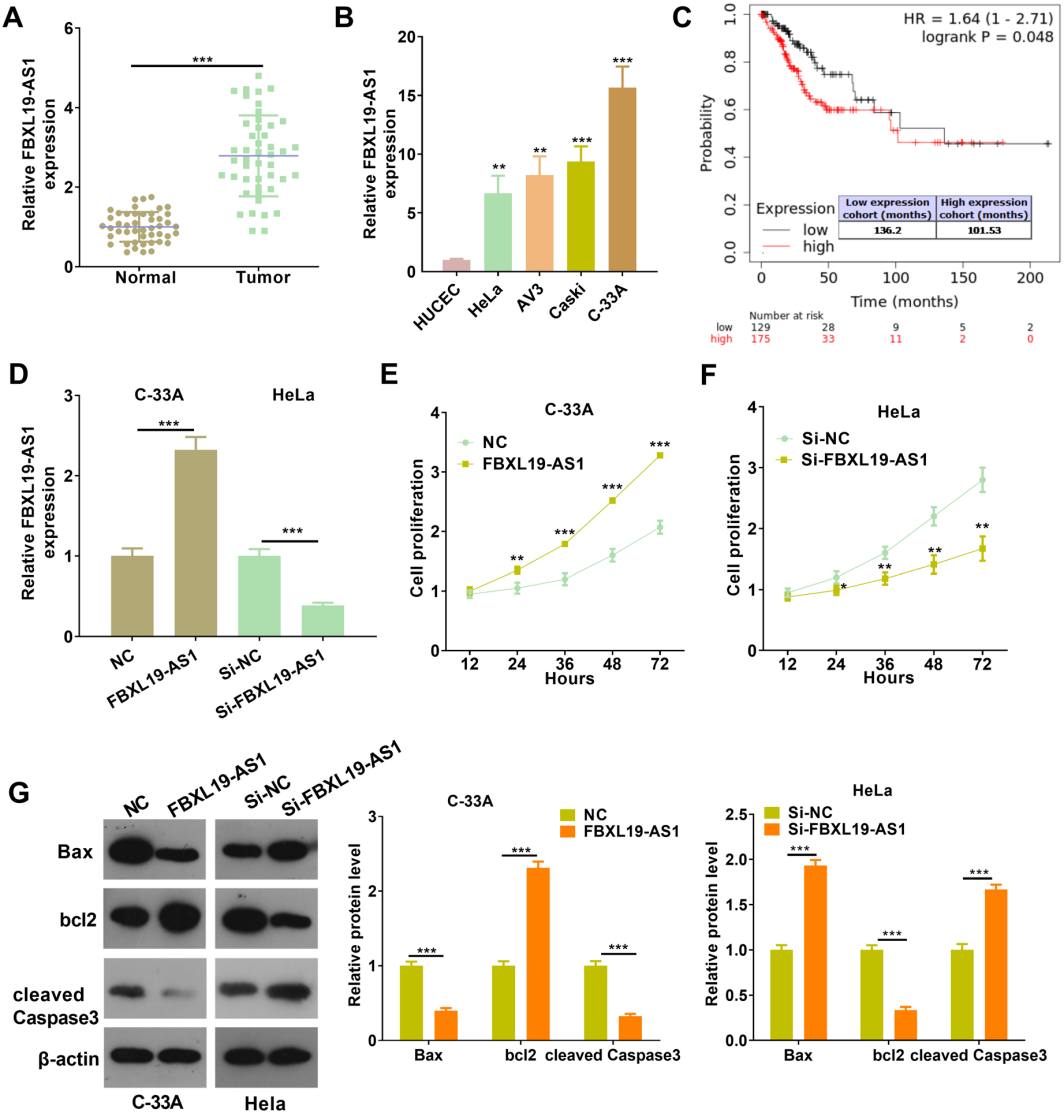
FBXL19-AS1 expression in cervical cancer tissues and its regulation on the proliferation and apoptosis of cervical cancer cells. **A** RT-PCR for detection of FBXL19-AS1 expression in 46 cases of CC tissues and adjacent normal tissues. **B** RT-PCR detection of FBXL19-AS1 expression in Human healthy cervical cells (HUCEC) and human cervical cancer cells (HeLa, Caski, C-33 A, AV3), **p < 0.01, ***p < 0.001 vs. HUCEC group; **C** KM plotter (http://kmplot.com/analysis/) was used for the analysis of FBXL-19-AS1 expression in the overall survival of CC patients. **D** C-33 A and HeLa cells were used for the cell model of FBXL19-AS1 overexpression and downregulation, and RT-PCR was used for detection of FBXL19-AS1 expression. **E**, **F** The effect of FBXL19-AS1 on the proliferation of C-33 A and HeLa cells was detected via CCK-8 assay, *p < 0.05, **p < 0.01, ***p < 0.001 vs. NC or si-NC group; **G** Western blot taken to detect the changes of apoptosis protein after FBXL19-AS1 regulation. ***p < 0.001 (n = 3)

To explore the potential role of FBXL19-AS1 in CC, RT-PCR was used to detect the level of FBXL19-AS1 in CC tissues and cells. The results indicated that FBXL19-AS1 was notably increased in CC tissues compared with that of adjacent normal tissues (*p* < 0.05, Fig. 1A) and CC cells compared with HUCEC cells (*p* < 0.05, Fig. 1B). The higher level of FBXL19-AS1 was associated with poorer differentiation of CC and the higher TNM stages (Table 1). Through KM plotter analysis (http://kmplot.com/analysis/), we found that higher FBXL-19-AS1 predicts poorer overall survival in CC tissues (Fig. 1C). Hence, those results indicated that FBXL19-AS1 might be an oncogene in CC. Next, gain- and loss-of functional assays of FBXL19-AS1 were conducted on C-33 A and HeLa cells, respectively (*p* < 0.05, Fig. 1D). The proliferation and apoptosis of the CC cells were tested. The results of CCK8 assay illustrated that the proliferation capacity of over-expressed FBXL19-AS1 cells was increased (compared with the NC group), and the proliferation capacity decreased after FBXL19-AS1 knockdown (compared with the si-NC group) (*p* < 0.05, Fig. 1E, F). Western blot assay was performed to detect apoptosis-related proteins. When FBXL19-AS1 was over-expressed, Bax and Caspase-3 were down-regulated while Βcl2 was upregulated. After knocking down FBXL19-AS1, Bax and cleaved Caspase-3 were upregulated while Bcl2 was down-regulated (*p <* 0.05, Fig. 1G). Therefore, it was deduced that FBXL19-AS1 was an unfavorable factor for CC diagnosis and functioned as an oncogene by promoting cell proliferation and inhibiting apoptosis.

**Table 1:** Relationship between FBXL19-AS1 expression level and clinical characteristics in tissue samples from patients with cervical cancer (CC)

Characteristics	Patients	Expression of FBXL19-AS1		*p*-value
		Low-FBXL19-AS1	High-FBXL19-AS1	
Total	46	23	23	
Age (years)				0.760
< 65	14	8	9	
≥ 65	32	15	14	
Differentiation				0.0382*
Middle or low	21	7	14	
High	25	16	9	
TNM stage				0.0219*
I~II	33	20	13	
III ~ IV	13	3	10	
Lymphatic metastasis				0.3457*
Yes	25	11	14	
No	21	12	9	

*p < 0.05, the difference was statistically valuable

### FBXL19-AS1 promoted metastasis and EMT of CC cells

**Fig. 2:**
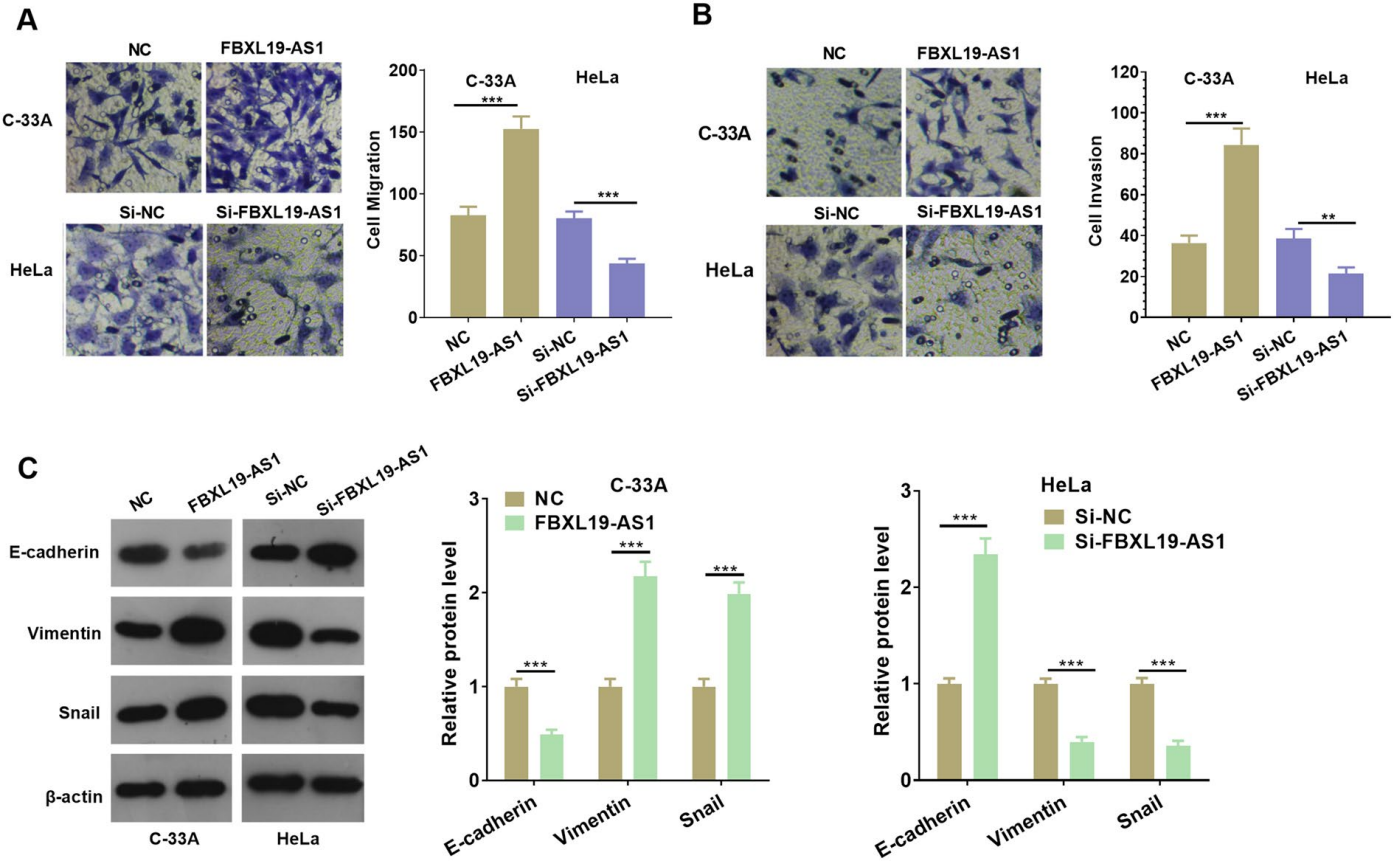
FBXL19-AS1 promoted cervical cancer cell migration, invasion and EMT. **A**, **B** Transwell detection of CC cell migration (**A**) and invasion (**B**) after regulation of FBXL19-AS1; **C** After regulation of FBXL19-AS1, the expressions of EMT-related markers (including E-cadherin, Vimentin and Snail) were detected by Western blot. **p < 0.01, ***p < 0.001 (n = 3)

**Fig. 3:**
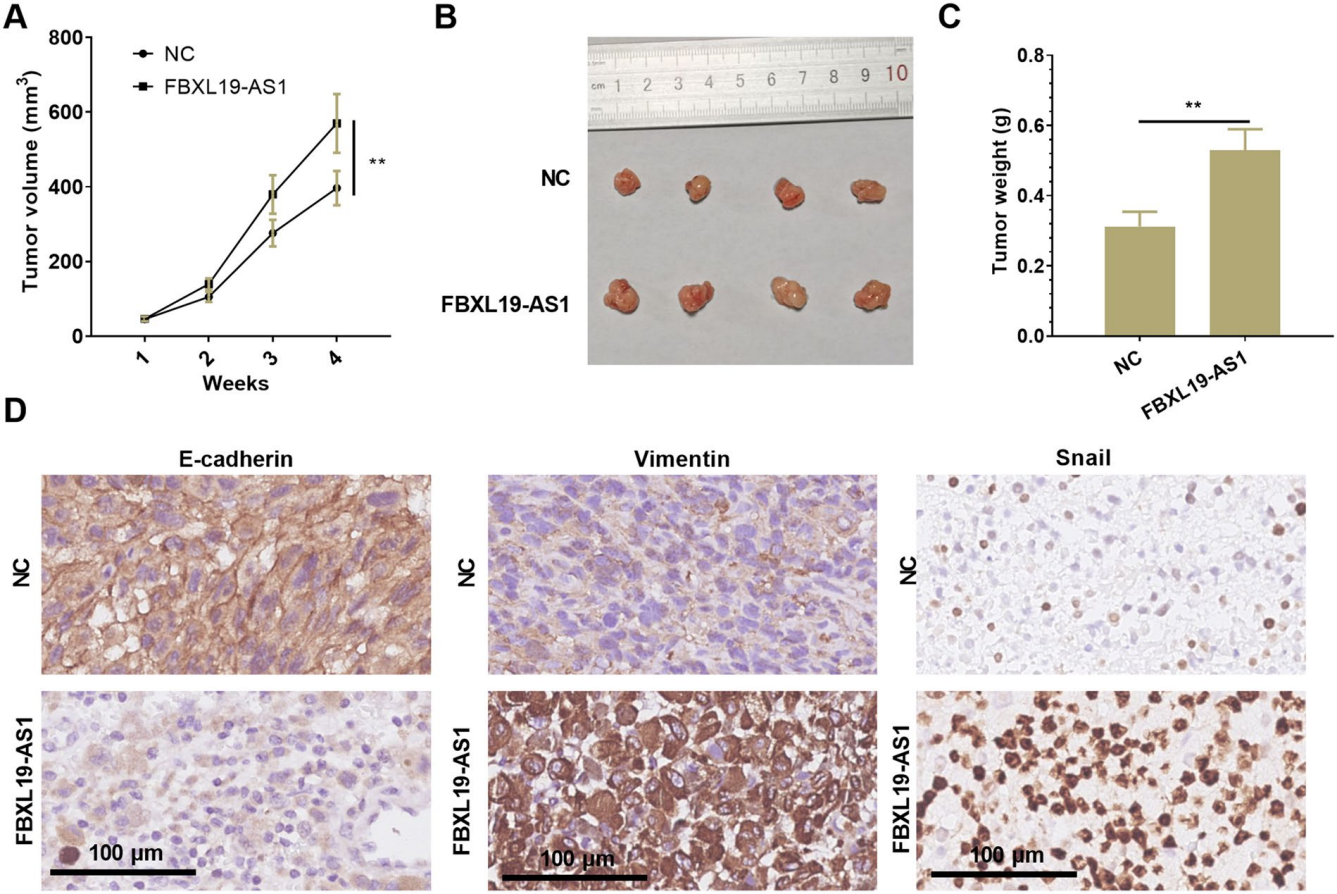
FBXL19-AS1 enhanced C-33 A cell growth and EMT in vivo. C-33 A cells were transfected with FBXL19-AS1 overexpression plasmids and then subjected to in-vivo experiment in nude mice. **A** Tumor volume was calculated every week. **B** Tumor image at the fourth week. **C** Tumor weight. **D** IHC was conducted to detect E-cadherin, Vimentin and Snail in the formed tissues. **p < 0.01 (n = 4)

The Transwell experiment was carried out to explore the effect of FBXL19-AS1 on the migration and invasion of CC cells. The experimental results demonstrated that the migration and invasion were remarkably enhanced after over-expressing FBXL19-AS1, while the opposite results were found after FBXL19-AS1 knockdown *(p <* 0.05, Fig. 2A, B). Moreover, as epithelial-mesenchymal transition (EMT) is a vital factor in mediating CC metastasis, the EMT markers of E-cadherin, Vimentin and Snail were examined by Western blot. The results showed that compared with the control group, E-cadherin expression was decreased after FBXL19-AS1 overexpression, and Vimentin and Snail expressions were upregulated. After knocking down FBXL19-AS1, E-cadherin expression was increased, while Vimentin and Snail expressions were down-regulated (*p* < 0.05, Fig. 2C). Furthermore, we performed in vivo experiment to confirm the function of FBXL19-AS1. We found the tumor growth was significantly enhanced following FBXL19-AS1 overexpression (Fig. 3A–C). In addition, the Vimentin and Snail expressions were remarkedly enhanced while E-cadherin was reduced in the FBXL19-AS1 compared with that of the NC group (Fig. 3D). The above results suggested that FBXL19-AS1 promoted the growth and metastasis of CC cells via aggravating the migration, invasion and EMT of them.

### FBXL19-AS1 targeted miR-193a-5p

**Fig. 4:**
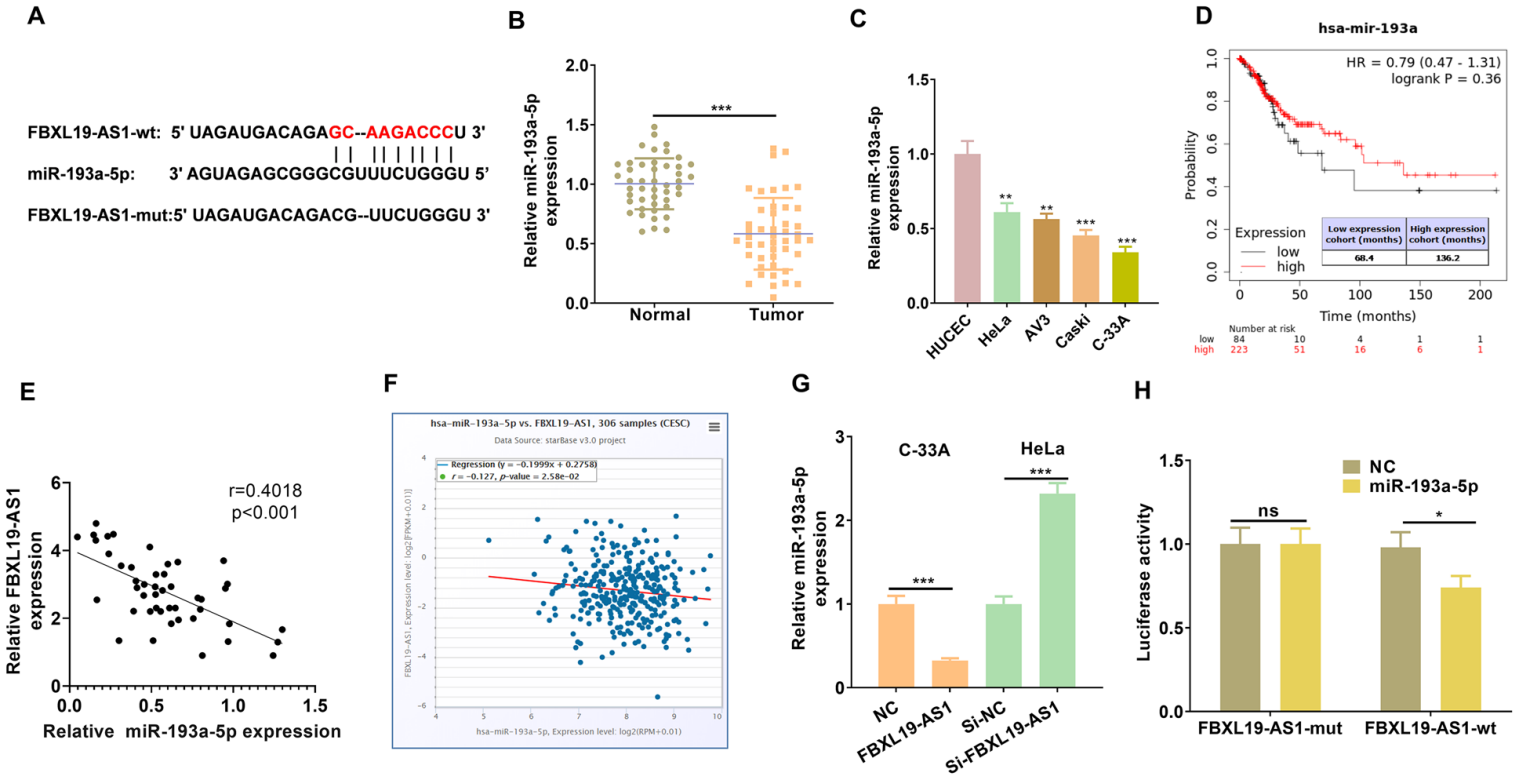
FBXL19-AS1 targeted miR-193a-5p. **A** The binding sites between FBXL19-AS1 and miR-193a-5p was predicted by Starbase (http://Starbase.sysu.edu.cn/); **B**, **C** RT-PCR experiment was employed to detect miR-193a-5p expression in 46 cases of CC tissues and CC cells, **p < 0.01, ***p < 0.001 vs. HUCEC group. **D **KM plotter (http://kmplot.com/analysis/) was used for the analysis of miR-193a-5p expression in the overall survival of CC patients. **E** Liner regression analysis of the FBXL19-AS1 and miR-193a-5p expression in CC tissues. **F** Starbase (http://Starbase.sysu.edu.cn/) showed that FBXL19-AS1 and miR-193a-5p expression in CC tissues were negatively associated. **G** MiR-193a-5p expression after up- or down-regulating FBXL19-AS1 was evaluated via RT-PCR; **H** Dual luciferase activity test was used for the confirmation of the binding relationship between FBXL19-AS1 and miR-193a-5p. nsP > 0.05, *p < 0.05, **p < 0.01, ***p < 0.001 (n = 3)

By browsing the online Starbase database (http://Starbase.sysu.edu.cn/) [25], the base complementary binding sites between FBXL19 AS1 and miR-193a-5p were found (Fig. 4A). By conducting RT-PCR, it was found that miR-193a-5p expression was considerably down-regulated in CC tissues (compared with normal tissues) (*p* < 0.05, Fig. 4B) and CC cells (compared with HUCEC cells) (*p* < 0.05, Fig. 4C). Besides, by analyzing the relationship between miR-193a-5p expression and the outcome of CC patients via KM plotter (http://kmplot.com/analysis/), we found that the CC patients with lower level of miR-193a-5p had worse overall survival rate (Fig. 4D). At the same time, we found that miR-193a-5p had a negative relationship with FBXL19-AS1 in terms of their expressions in CC tissues (Fig. 3E, F). Additionally, miR-193a-5p expression was notably decreased after FBXL19-AS1 over-expression, while it was upregulated after FBXL19-AS1 knockdown (*p* < 0.05, Fig. 3G). To further confirm the targeting relationship between the two, we carried out luciferase reporter gene experiment. The results represented that miR-193a-5p had no obvious effect on the luciferase activity of the FBXL19-AS1-mut vector that was mutant at the binding sites with miR-193a-5p, but miR-193a-5p inhibited the luciferase activity of the FBXL19-AS1-wt vector (*p* < 0.005, Fig. 3H). Based on the above results, we believed that FBXL19-AS1 had a targeted binding relationship with miR-193a-5p and negatively regulated miR-193a-5p expression.

### miR-193a-5p inhibited the malignant behaviors of CC cells

**Fig. 5:**
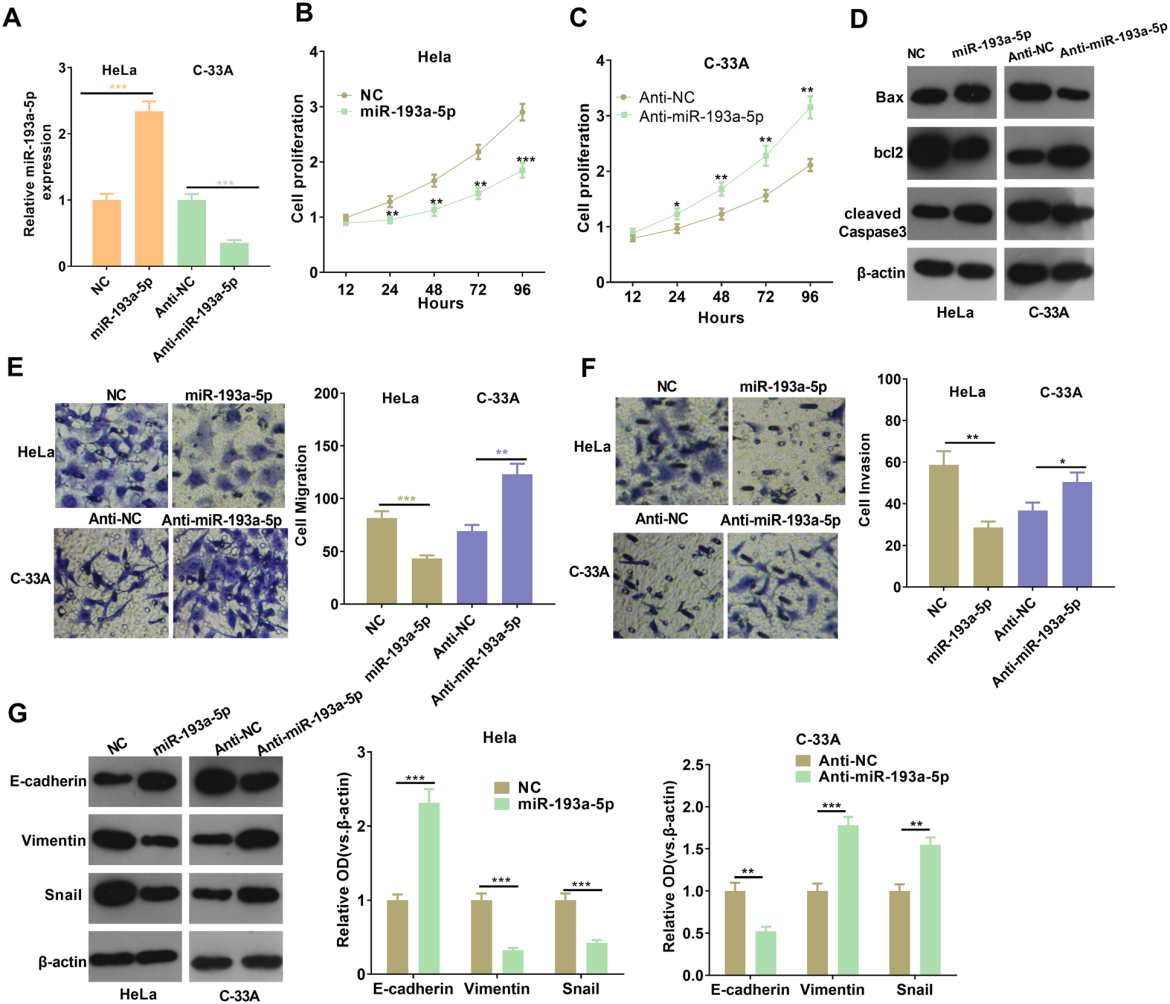
MiR-193a-5p limited the malignant behaviors of CC cells. **A** MiR-193a-5p expression in CC cells (Hela and C-33 A) transfected with miR-193a-5p mimics or inhibitors was detected by RT-PCR; **B**, **C** CCK-8 was used to detect the effects of miRs-193a-5p on CC cell proliferation, *p < 0.05, **p < 0.01, ***p < 0.001 vs.NC group or anti-NC group. **D** Western blot was used for the detection of apoptotic proteins; **E**, **F** Transwell was used for the detection of the migration and invasion ability of CC cells; **G** The expressions of EMT-related markers in CC cells after transfection with miR-193a-5p mimics or inhibitors were detected by western blot. *p < 0.05, **p < 0.01, ***p < 0.001 (n = 3)

To further verify the role of miR-193a-5p in CC, we transfected miR-193a-5p mimics or miR193a-5p inhibitors in HeLa and C-33 A cells respectively (*p* < 0.05, Fig. 5A). Besides, CCK-8 assay and Western blot were performed to examine cell proliferation and apoptosis. The results illustrated that, compared with the NC group, the proliferative level of HeLa cells reduced miR-193a-5p upregulation, and the apoptosis level was increased, while inhibition of miR-193a-5p enhanced cell proliferation and dampened apoptosis of C-33 A cells (*p* < 0.05, Fig. 5B–D). In addition, the Transwell results illustrated that the migration and invasion levels of CC cells in the miR-193a-5p mimics group were down-regulated, while those in the anti-miR-193a-5p group were upregulated (*p* < 0.05, Fig. 5E, F). Western blot results showed that E-cadherin expression in miR-193a-5p mimics group was upregulated, and Vimentin and Snail expressions were down-regulated, while anti-miR-193a-5p group showed the opposite trends (*p* < 0.05, Fig. 5G). Combined with the above results, we speculated that miR-193a-5p inhibited CC progress as an anti-tumor gene.

### FBXL19-AS1 overexpression limited the anti-tumor effects of miR-193a-5p

**Fig. 6:**
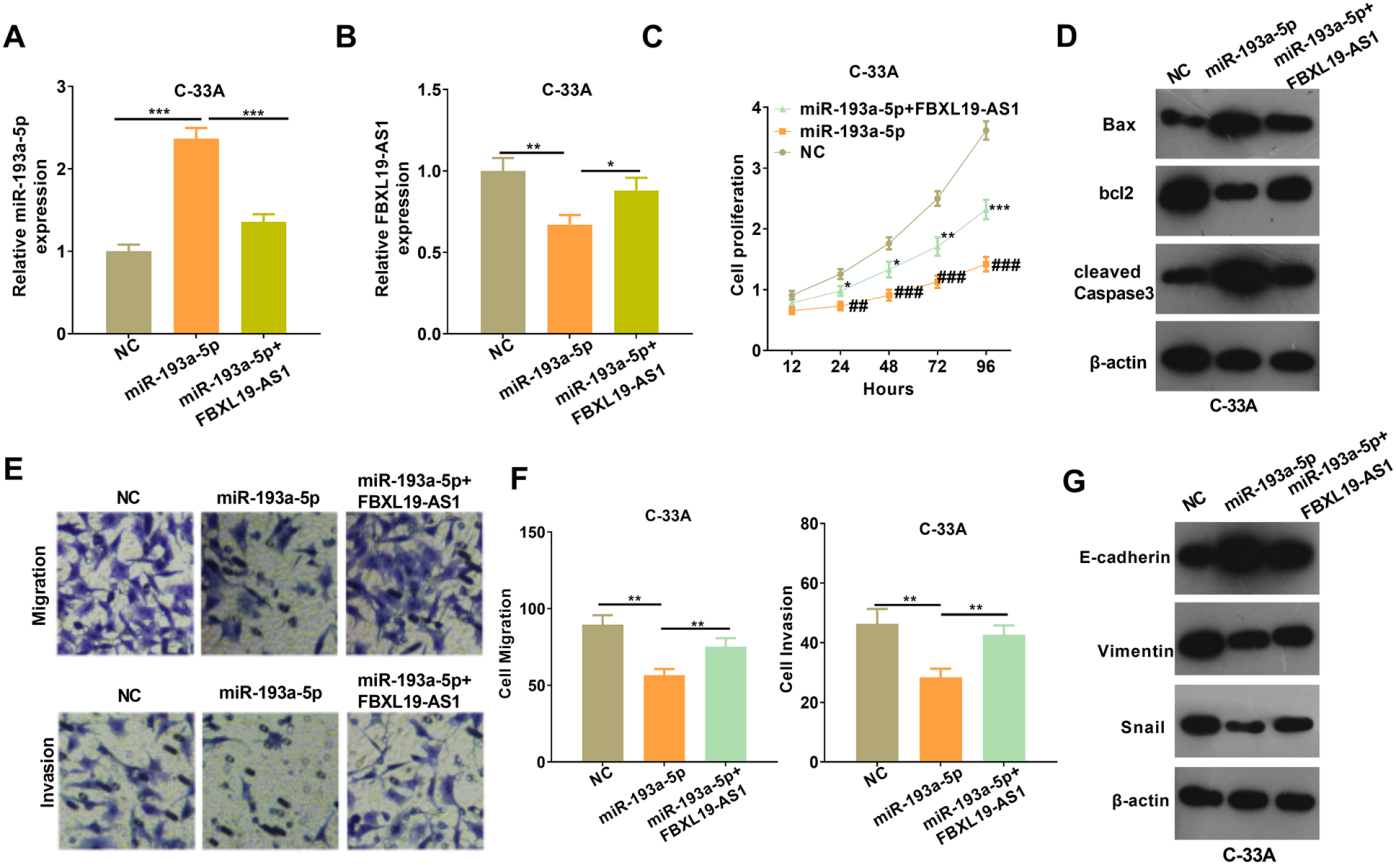
FBXL19-AS1 inhibited the effects of miR-193a-5p on CC cells. **A**, **B** C-33 A cells were transfected with miR-193a-5p mimics and/or FBXL19-AS1 overexpressing plasmids, then RT-PCR was conducted to examine the level of miR-193a-5p (**A**) and FBXL19-AS1 (**B**); **C** CCK8 test was performed to detect cell proliferation ability, ^##^*p* < 0.01 and ^###^*p* < 0.001 vs. NC group, *p < 0.05 and **p < 0.01 vs. miR-193a-5p group; **D** Western blot test to measure apoptosis-related proteins; **E**, **F** Transwell was applied to examine cell migration and invasion ability; **G** Western blot test to determine the expression of EMT-related markers in each group. *p < 0.05, **p < 0.01, ***p < 0.001 (n = 3)

Aiming on the exploration of how FBXL19-AS1/miR-193a-5p axis influences tumor progression, we co-transfected C-33 A cells with FBXL19-AS1 overexpressing plasmids and miR-193a-5p mimics. The expressions of FBXL19-AS1 and miR-193a-5p was detected via RT-PCR. The results showed that FBXL19-AS1 notably attenuated miR-193a-5p up-regulation induced by the transfection of miR-193a-5p mimics. On the other hand, FBXL19-AS1 was notably decreased when transfected with miR-193a-5p mimics (*p* < 0.05, Fig. 6A, B). The results of CCK8 experiment showed that the addition of FBXL19-AS1 increased the cell proliferation capacity compared with that in miR-193a-5p group (*p* < 0.05, Fig. 6C). Next, the results of western blot indicated that compared with the miR-193a-5p group, the Bax and cleaved Caspase-3 levels in the FBXL19-AS1 overexpression group were attenuated while Bcl2 level was elevated (Fig. 6D). Furthermore, FBXL19-AS1 upregulation significantly accelerated the migration, invasion and EMT of C-33 A-cells compared with miR-193a-5p group (*p* < 0.05, Fig. 6E–G). Collectively, the above results suggested that FBXL19-AS1 inhibited the inhibitive effects of miR-193a-5p on CC cells.

### miR-193a-5p targeted COL1A1

**Fig. 7:**
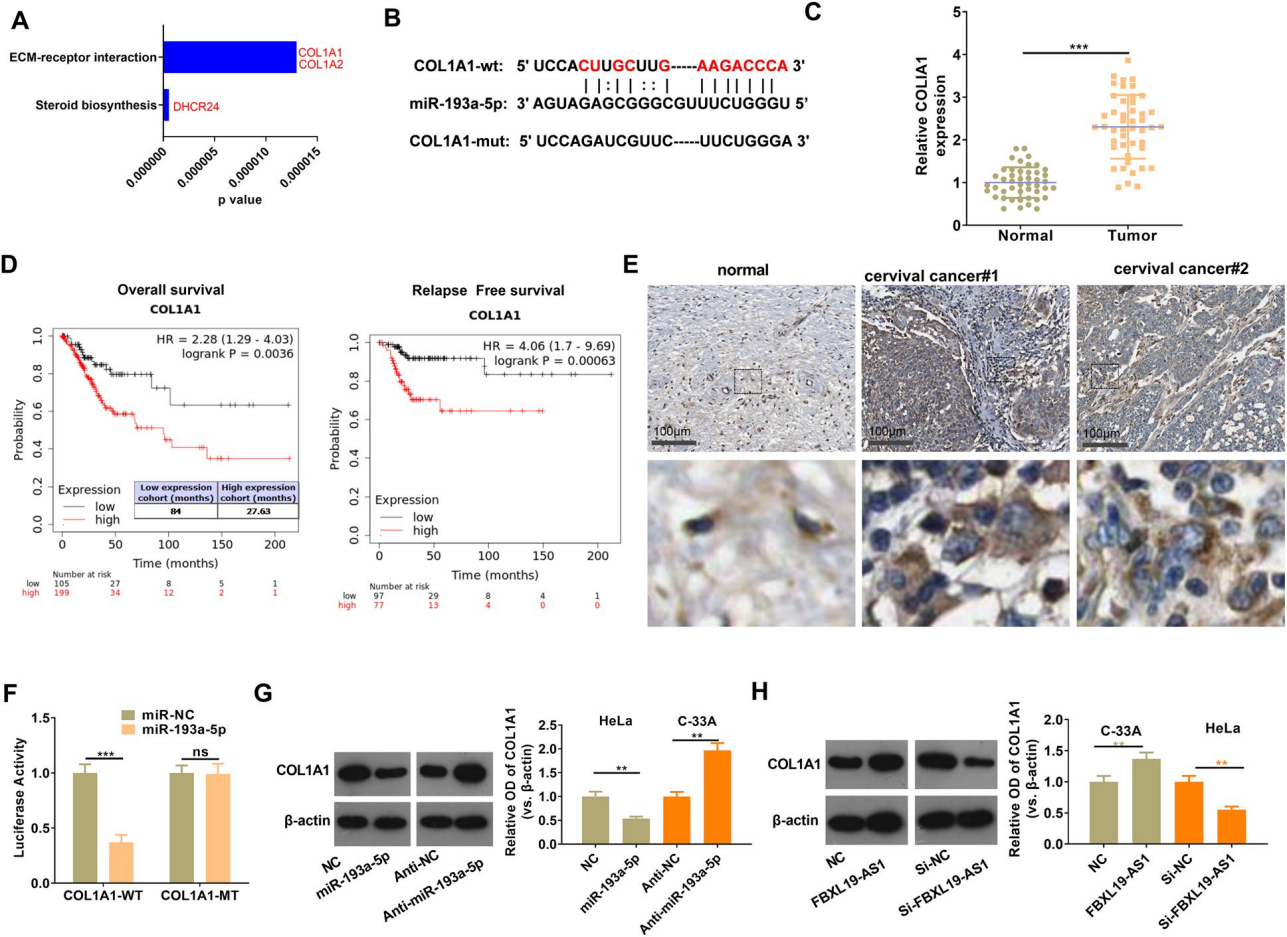
miR-193a-5p targeted COL1A1. **A **The downstream pathways modulated by miR-193a-5p was analyzed via mirPath v.3 (http://snf-515788.vm.okeanos.grnet.gr/index.php?r=mirpath). **B** TargetScan database (http://www.targetscan.org/vert_72/) was used for predicting the binding sites between miR-193a-5p and COL1A1. **C** RT-PCR was performed to test COL1A1 expression in CC tissues. **D** The diagnostic significance of COL1A1 in CC was analyzed via KM plotter (http://kmplot.com/analysis/). **E** The expression of COL1A1 in normal cervical tissues and cervical cancer tissues was analyzed in The Human Protein Alas (https://www.proteinatlas.org/). **F** luciferase reporter gene experiment was conducted to confirm the targeting relationship between miR-193a-5p and COL1A1. **G**, **H** Western blot was used for the detection of COL1A1 in CC cells with gain and loss of miR-193a-5p (**G**) and FBXL19-AS1 (**H**) expression. ns *p* > 0.05, **p < 0.01, ***p < 0.001 (n = 3)

Next, we further analyzed the downstream pathways modulated by miR-193a-5p via mirPath v.3 (http://snf-515788.vm.okeanos.grnet.gr/index.php?r=mirpath). Two candidate KEGG pathways were found, namely the Steroid biosynthesis and the ECM-receptor interaction, and there were three candidate genes potentially regulated by miR-193a-5p (Fig. 7A). Next, TargetScan database (http://www.targetscan.org/vert_72/) was used for predicting the binding sites of the three genes with miR-193a-5p. Interestingly, only COL1A1 was found to have complementary binding sites with miR-193-a-5p (Fig. 7B). Following that, RT-PCR was performed to verify COL1A1 expression in CC tissues, and the results illustrated that COL1A1 expression was significantly upregulated in CC tissues compared with that in the normal tissues (*p* < 0.05, Fig. 7C). By analyzing the diagnostic significance of COL1A1 in CC via KM plotter (http://kmplot.com/analysis/), we found that higher level of COL1A1 predicts poorer overall survival and Relapse Free survival of CC patients (*p* < 0.05, Fig. 7D). Besides, we also found that the COL1A1 was lowly expression in normal cervical tissues but obviously upregulated in CC tissues (COL1A1 was mainly located in the cytoplasm CC cells) (Fig. 7E). Next, by conducting luciferase reporter gene experiment, we confirmed that miR-193a-5p targeted at the 3΄UTR of COL1A1 mRNA (Fig. 7F). Moreover, gain and loss of miR-193a-5p and FBXL19-AS1 revealed that miR-193a-5p inhibited COL1A1 while FBXL19-AS1 promoted COL1A1 expression in CC cells (Fig. 7G, H). Thus, the FBXL19-AS1/miR-193a-5p could modulate CC progression potentially through COL1A1, which was a promising oncogene in CC.

### Inhibition of COL1A1 weakened CC cell proliferation and metastasis

**Fig. 8:**
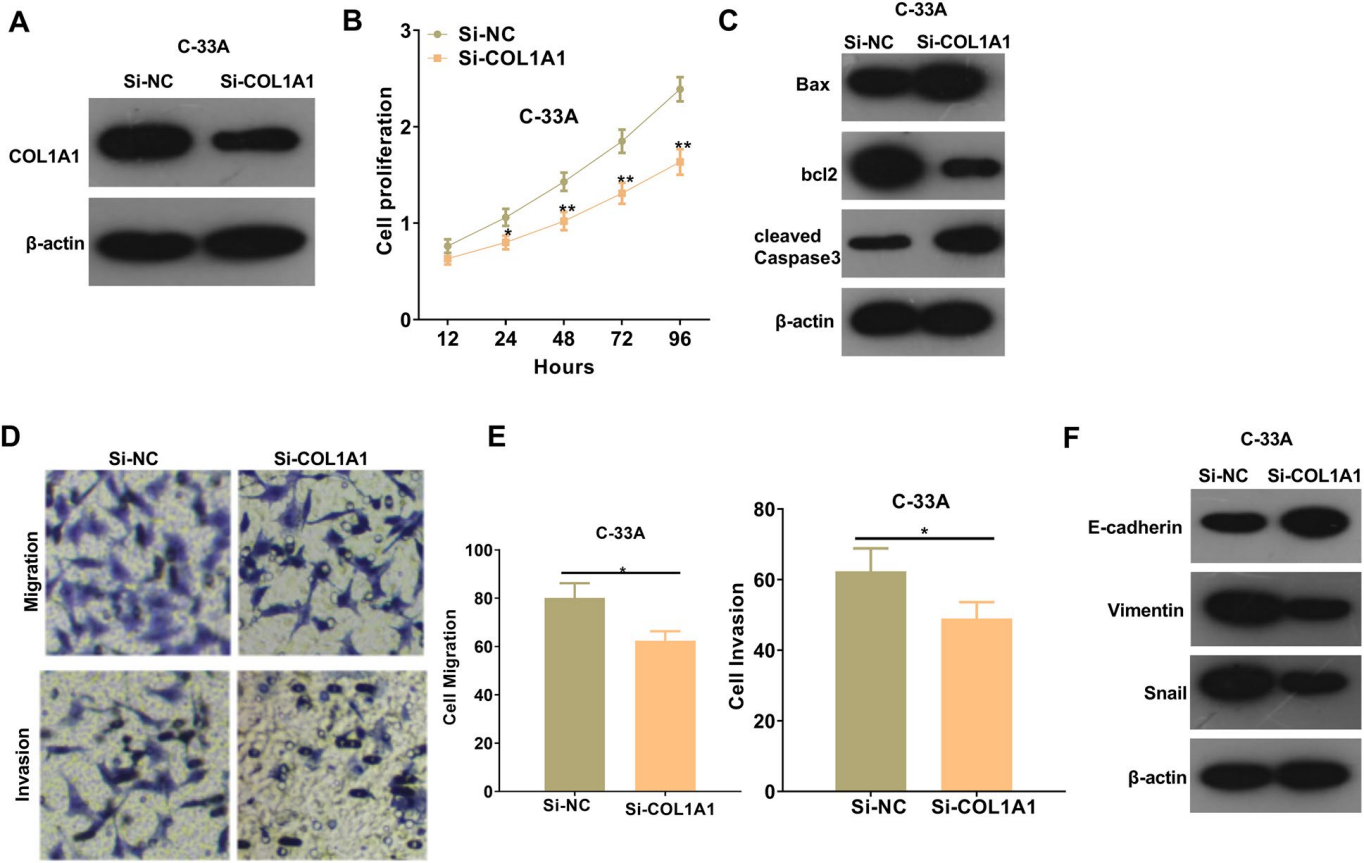
Inhibition of COL1A1 attenuated CC cell proliferation and metastasis. **A** C-33 A cells were transfected with si-NC or si-COL1A1 and Western blot was used for the detection of COL1A1 protein expression; **B** CCK-8 assay was taken to measure cell proliferation changes after COL1A1 knockdown, *p < 0.05 and **p < 0.01 vs. si-NC group; **C** Apoptosis protein expression after COL1A1 knockdown treatment was evaluated by western blot; **D**, **E** Transwell was applied to detect the ability of cancer cell migration and invasion after COL1A1 knockdown; **F** Western blot was taken to measure the expression of relevant EMT-related markers after COL1A1 knockdown. *p < 0.05

To further explore the functions of COL1A1 in CC development, a COL1A1 knockdown cell model was constructed (Fig. 8A). By detecting the cell proliferation via CCK8 assay, we found that COL1A1 knockdown resulted in a considerable decrease of cell proliferation (*p* < 0.05, Fig. 8B). Western blot results illustrated that knockdown of COL1A1 upregulated Bax and cleaved Caspase-3 expressions and inhibited Bcl2 expression (Fig. 8C). Meanwhile, COL1A1 downregulation also led to the attenuation of migration, invasion and EMT of CC cells (Fig. 8D–F). In summary, COL1A1 functions as an oncogene in CC through modulating the proliferation, apoptosis, migration, invasion and EMT of CC cells.

## Discussion

**Fig. 9:**
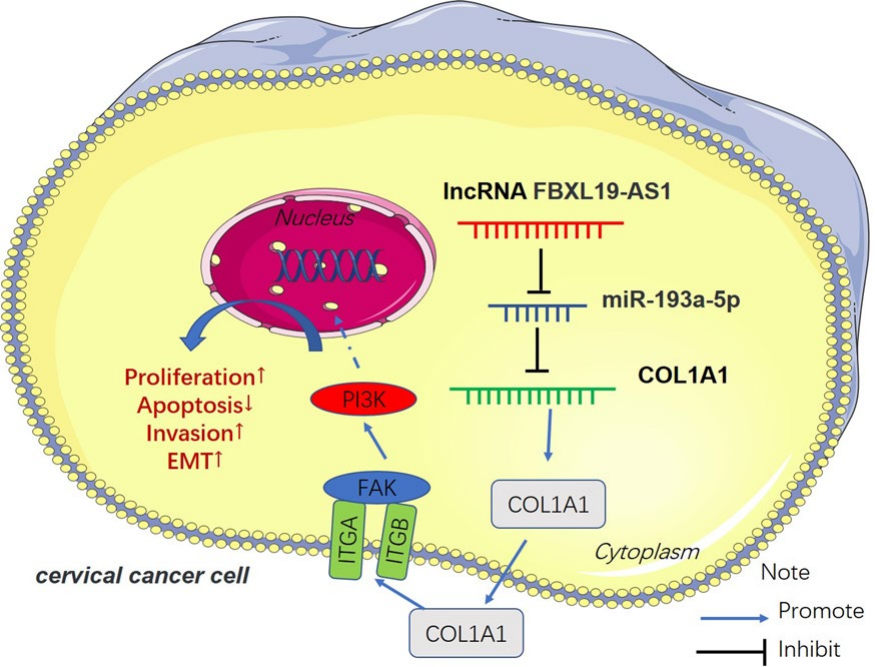
The mechanism diagram. FBXL19-AS1 was overexpressed in CC. It functioned as a “ceRNA” by sponging miR-193a-5p, thus upregulating COL1A1 and accelerating CC cell growth, metastasis, EMT and inhibit apoptosis

Cervical cancer is one severe tumor in the female reproductive system. Due to its high morbidity and mortality, women of all ages are susceptible to the disease, especially middle-aged women, which seriously affects womens’ mental health and social life [26, 27]. Hence, to identify the molecular mechanisms and therapeutic targets is necessary for cervical cancer occurrence and development. The present study showed that FBXL19-AS1 is upregulated in CC tissues and acts as an unfavorable predictor of CC patents. Moreover, our functional and mechanistical experiments indicated that FBXL19-AS1 promotes CC development via mediating miR-193a-5p/COL1A1 axis (Fig. 9).

Unlimited growth and being prone to metastasis belong to the two crucial factors of tumor progression, which are the same for CC. Therefore, many strategies have been applied in the treatment of CC via modulating the apoptosis and metastasis of CC [28, 29]. Though lncRNAs are limited with gene coding abilities, multiple lncRNAs have been found to be dysregulated in CC and have obvious effects on the malignant behaviors of CC cells. For example, lncRNA SNHG1 was upregulated in CC tissues and cell lines and overexpression of SNHG1 promotes the proliferation, migration and invasion of CC cells [30]. LncRNA SRA, which participates in cervical cancer cell proliferation and migration, can be used as a potential therapeutic target and prognostic marker for cervical cancer [31]. As a novel lncRNA, FBXL19-AS1 was found to play a role in multiple tumors such as osteosarcoma [8], breast cancer [7] and lung cancer [32]. Surprisingly, this study found that FBXL19-AS1 is overexpressed in CC tissues and cells. Besides, the upregulated FBXL19-AS1 predicts poorer survival of CC patients. Furthermore, it promoted CC cell proliferation, migration and invasion. Those results confirmed that FBXL19-AS1 is a referable molecular target for CC treatment and prognosis.

Epithelial-mesenchymal transition (EMT) is a crucial process in many types of human epithelial carcinoma including CC. Losing the epithelial properties (marked by E-cadherin) and gaining mesenchymal characteristics (marked by Vimentin, N-cadherin, Snail) are two main features of EMT [33, 34]. Additionally, EMT is also an important process in the invasion and metastasis of human cervical carcinoma [35]. Interestingly, many lncRNAs are found to affect the EMT of CC cells. For instance, downregulation of lncRNA AFAP1‑AS1 not only suppressed the migration and invasion of cervical cancer cells, but also inhibited epithelial‑mesenchymal transition (EMT)‑related genes [36]. Interestingly, FBXL19-AS1 has also been proved to modulate the EMT process of cancers. For example, FBXL19-AS1 upregulation enhanced the migration, invasion and EMT of non-small cell lung cancer [37] and breast cancer [38]. Here, we also evaluated the EMT of CC cells under selective regulation of FBXL19-AS1 level. Our data showed that FBXL19-AS1 overexpression led to higher level of Vimentin and Snail but less expression of E-cadherin. Therefore, FBXL19-AS1 could regulate the metastasis of CC cells via enhancing EMT.

MiRNA participates in the regulation of target genes through complementary base pairing, thus affecting the pathophysiological changes of diseases [39, 40]. MiRNA exists stably in the blood circulation and has great application value in the early diagnosis, stage determination and prognosis assessment of CC, besides, miRNA also exerts significant effects on the malignant behaviors of CC. Taking miR-497-5p as an example, it directly targeted the multi-comb chromosome box 4 (CBX4) and inhibited CC cell proliferation [41]. MiR-3647-5p has been shown to target AGR2 to limit CC cell proliferation and promote apoptosis [42]. Studies have found that miR-193a-5p has an anti-cancer effect in various tumors such as osteosarcoma [14] and hepatocellular carcinoma [43]. Interestingly, our study also showed that miR-193a-5p was decreased in CC tissues and limited cell proliferation and migration, suggesting that miR-193a-5p plays an anti-cancer role in CC.

Type I collagen is an important member of the collagen family which is a key structural component of the extracellular matrix. COL1A1 and COL1A2 are two members of Type I collagen, interestingly, both of COL1A1 and COL1A2 are dysregulated in several cancers and involved in tumor invasion and progression [19, 44]. For example, in breast cancer, the increased COL1A1 level was associated with poor survival, especially in patients with ER + breast cancer. Knockdown of COL1A1 inhibited metastasis and EMT of breast cancer cells [45]. Besides, COL1A1 is upregulated in colorectal cancer (CRC) and promoted CRC cell migration via WNT/PCP signaling pathway [46]. In our study, we discovered that COL1A1 was over-expressed in CC tissues and cells, and knocking down COL1A1 considerably attenuated cell proliferation, migration and invasion and accelerated cell apoptosis. Thus, COL1A1 was proven as an oncogene in CC.

Recently, the lncRNA-miRNA-mRNA network has aroused many researchers’ interests, especially in tumor progression. Many lncRNAs have been found to function as a competitive endogenous RNA (ceRNA) via targeting miRNAs, and inhibit those miRNAs level and/or reduce the activity of those miRNAs in cells [47]. In cancer cells and tissues, miRNAs might be more susceptible to degradation due to the interaction with the aberrantly expressed ncRNAs, especially lncRNAs, thereby regulating important cancer-related genes’ expression [48]. miRNA targets at the 3΄UTR of its target gene mRNA. Thus, lncRNA indirectly promotes gene expression [49, 50]. For instance, lncRNA NEAT1 upregulated the expression of EZH2 and promoted breast cancer growth by inhibiting miR-101 expression [51]. In CC, lncRNA DANCR regulated ROCK1 expression by competitively binding to miR-335-5p [52]. Here, we found that there are binding sites between FBXL19-AS1 and miR-193a-5p by analyzed through Starbase. Functionally, FBXL19-AS1 overexpression inhibited miR-193a-5p expression and also dampened the anti-tumor effects of miR-193a-5p by conducting rescue experiment. We supposed that FBXL19-AS1 not only sponges miR-193a-5p, but also leads to its degradation. In addition, miRpath v3.0 showed that COL1A1 was a candidate gene of miR-193a-5p. Also, TargetScan database showed a binding site between miR-193a-5p and COL1A1, which encouraged us to further explore whether FBXL19-AS1 works in promoting cancer by indirectly regulating COL1A1 expression. Our data indicated that COL1A1 expression was upregulated following FBXL19-AS1 over-expression and downregulated when miR-193a-5p was upregulated. Thus, FBXL19-AS1 upregulated COL1A1 expression by inhibiting miR-193a-5p (Fig. 9).

To sum up, the upregulated expression of FBXL19-AS1 in CC may serves as a reference index for CC diagnosis. Moreover, FBXL19-AS1 upregulates COL1A1 by targeting miR-193a-5p to accelerate CC cell growth, metastasis, EMT and inhibit apoptosis. The FBXL19-AS1/miR-193a-5p /COL1A1 axis plays a significant role by modulating the development of CC. However, more experiments such as in vivo assays and clinical experiments are needed to further confirm the biofunctions of this novel axis in CC progression.

## Data Availability

The data sets used and analyzed during the current study are available from the corresponding author on reasonable request.
